# The Right of Petition

**Published:** 1873-03

**Authors:** 


***The Right of Petition.***

In the war against one of the Municipal Boards, recently inaugurated in this much governed city, the Board of Trade, by its petition to the Legislature, for the abolition of one board, has set an example to the medical profession, which might be followed with great advantage by a similar petition, for the abolition of another, the Board (miscalled) of Health. If there is a single respectable physician in Chicago, outside of the Health Department, who can be found to sustain this monument of inefficiency, we shall be most happy to hear from him in its defense. We have been at some pains to ascertain the opinions of professional men upon this subject, and find the unanimous verdict to be one of condemnation, (varied, only, as to orthography.)

The people look to their physicians for advice in matters pertaining to health, and it is not enough for each to care for the health of his patients individually. Something he owes to society in its aggregate. The great mass of the populace is incapable of protecting its own health, and for this must the medical profession speak, when governments neglect their duty.

The condition of our city, in view of prospective danger to public health during the coming summer, is truly alarming, and demands the immediate action of the profession, not only as guardians of public health, but as citizens, whose lives are endangered, as are the lives of those who are near and dear to them. The Chicago public, with all its faults, is immensely charitable, long suffering and forgiving. It suffered the large mortality of 1872, costing, for weeks together, the life of one of its citizens every thirty minutes, it forgave these disastrous results, to the Board of Health, pleading the general demoralization consequent upon the fire as its excuse. The fire is an incident of the past. Commerce, trade, the arts, have revived, have acquired increased strength from disaster, and flourish with a vigor unknown before.

The organization constituted for the preservation of the public health, has exhibited only an increase of vis inertiæ, with which to meet the increased demands upon its sanitary nescience and administrative and executive inability.

The JOURNAL has been, during nearly six years, a silent observer of the revolution of this municipal grindstone, and of the clumsy efforts at axe-grinding made thereupon. "Turn about is fair play," even in grinding axes, and it would seem to be the public's turn now. If the public will only examine this machinery, it will be found especially adapted to private, not to public uses; for such ends it was designed, and to such it has been diligently applied, from the beginning.

The time has arrived when silence is almost equivalent to participation in guilt. The time has come for the medical profession as a body to speak openly its true sentiments, in denunciation of this legislative abortion, this miscalled Board of Health, to counsel the public to petition the legislature to revoke the acts of its predecessor, to abate this greatest of all nuisances, which has so long been a burlesque upon sanitary science, a disgrace to the city, and has now become imminently dangerous to public health. H.

